# Patient-Derived Human Basal and Cutaneous Squamous Cell Carcinoma Tissues Display Apoptosis and Immunomodulation following Gas Plasma Exposure with a Certified Argon Jet

**DOI:** 10.3390/ijms222111446

**Published:** 2021-10-23

**Authors:** Fariba Saadati, Juliane Moritz, Julia Berner, Eric Freund, Lea Miebach, Iris Helfrich, Ingo Stoffels, Steffen Emmert, Sander Bekeschus

**Affiliations:** 1 ZIK Plasmatis, Leibniz Institute for Plasma Science and Technology (INP), Felix-Hausdorff-Str. 2, 17489 Greifswald, Germany; fariba.saadati@inp-greifswald.de (F.S.); juliane.moritz@inp-greifswald.de (J.M.); julia.berner@inp-greifswald.de (J.B.); eric.freund@inp-greifswald.de (E.F.); lea.miebach@inp-greifswald.de (L.M.); 2 Department of Oral and Maxillofacial Surgery, Plastic Surgery, Greifswald University Medical Center, Ferdinand-Sauerbruch-Str., 17475 Greifswald, Germany; 3 Department of General, Visceral, Thoracic and Vascular Surgery, Greifswald University Medical Center, Ferdinand-Sauerbruch-Str., 17475 Greifswald, Germany; 4 Departments of Dermato-Oncology, Essen/Duisburg University Medical Center, Hufelandstr. 55, 45147 Essen, Germany; iris.helfrich@uk-essen.de (I.H.); ingo.stoffels@uk-essen.de (I.S.); 5 West-German Cancer Center, Essen/Duisburg University Medical Center, Hufelandstr. 55, 45147 Essen, Germany; 6 German Consortium for Translational Cancer Research, Essen/Duisburg University Medical Center, Hufelandstr. 55, 45147 Essen, Germany; 7 Department of Dermatology and Allergology of the Ludwig Maximilian University Hospital Munich, 80337 Munich, Germany; 8 Clinic for Dermatology and Venerology, Rostock University Medical Center, Strempelstr. 13, 18057 Rostock, Germany; steffen.emmert@med.uni-rostock.de

**Keywords:** chemokines, cold physical plasma, cytokines, reactive oxygen species, ROS, skin cancer

## Abstract

Reactive oxygen species (ROS) have been subject of increasing interest in the pathophysiology and therapy of cancers in recent years. In skin cancer, ROS are involved in UV-induced tumorigenesis and its targeted treatment via, e.g., photodynamic therapy. Another recent technology for topical ROS generation is cold physical plasma, a partially ionized gas expelling dozens of reactive species onto its treatment target. Gas plasma technology is accredited for its wound-healing abilities in Europe, and current clinical evidence suggests that it may have beneficial effects against actinic keratosis. Since the concept of hormesis dictates that low ROS levels perform signaling functions, while high ROS levels cause damage, we investigated herein the antitumor activity of gas plasma in non-melanoma skin cancer. In vitro, gas plasma exposure diminished the metabolic activity, preferentially in squamous cell carcinoma cell (SCC) lines compared to non-malignant HaCaT cells. In patient-derived basal cell carcinoma (BCC) and SCC samples treated with gas plasma ex vivo, increased apoptosis was found in both cancer types. Moreover, the immunomodulatory actions of gas plasma treatment were found affecting, e.g., the expression of CD86 and the number of regulatory T-cells. The supernatants of these ex vivo cultured tumors were quantitatively screened for cytokines, chemokines, and growth factors, identifying CCL5 and GM-CSF, molecules associated with skin cancer metastasis, to be markedly decreased. These findings suggest gas plasma treatment to be an interesting future technology for non-melanoma skin cancer topical therapy.

## 1. Introduction

Basal cell carcinoma (BCC) and squamous cell carcinoma (SCC) are highly prevalent skin cancers [1,2]. SCC is a relatively slow-growing skin cancer that can expand to the surrounding tissue and further into the body (metastasis). On the contrary, BCC rarely metastasizes but has invasive and destructive local growth [3]. Changes and diversity in the tumor microenvironment (TME) and the unknown nature of several other factors partially explain the failure of definitive treatment for these diseases in some cases [4,5]. Based on recent studies, the tumor microenvironment (TME) plays a significant role in tumor progression, response to treatment, growth, and the acquisition of the metastatic pattern by tumor cells [6]. In addition to all the classic strategies of tumors escaping the immune system responses, e.g., antigen expression reduction, resistance to cell lysis by the immune cells, and the expression and secretion of immune suppressor factors, the tumor also escapes the immune system under TME complexity [7,8]. Thus, looking for different treatment methods to target these tumors, especially in cases where resection is impossible, and examining the TME response to treatment is of utmost importance.

In recent decades, cold physical plasma has been used as a powerful tool for inhibiting growth and destroying cancer cells [9,10]. Numerous studies have demonstrated the physical plasma’s toxicity on different tumor entities, including glioblastoma [11,12] and skin [13,14], breast [15,16,17], colorectal [18,19], head and neck [20,21], and lung cancer [22,23]. The anticancer mechanism of physical plasma is complex, but it is assumed that the plethora of reactive oxygen species (ROS) generated by the plasma discharge affects cell functions by disrupting the intracellular redox balance and membrane antioxidant enzymes [24]. ROS are relatively short-lived molecules containing oxygen that are chemically active [25]. At supraphysiological concentrations, ROS damage membranes, DNA, mitochondria, and the endoplasmic reticulum, eventually leading to, e.g., apoptosis, necrosis, senescence, or autophagy [26,27]. Moreover, the mechanism of chemotherapy, photodynamic therapy, and radiotherapy are at least partially based on ROS generation and subsequent apoptosis induction [28,29]. Due to tumor cells’ strong reliance on physiological homeostasis with ROS, they are prone to perturbations in redox homeostasis and oxidative distress [30]. Importantly, such stress is also considered a central prerequisite in many types of chemotherapy [31]. 

Many cancers express a strong network of cytokines and chemokines as well as their receptors. For instance, tumor cells stimulate endothelial cells by chemokine and cytokine expression to loosen their contact inhibition for invasion and metastasis to other tissues [32]. In general, the immune system plays a pivotal and influential role in maintaining the body’s homeostasis and destroying tumors, in addition to its fundamental role in dealing with external and infectious agents [33]. Tumor cells can manipulate immune cells by, for instance, adapting their metabolic needs to ultimately enhance instead of halt cancer growth [34]. Hence, investigating the TME is of great importance in oncology.

In this study, the toxicity of a clinically certified atmospheric pressure argon plasma jet on skin cancer cells was investigated in vitro, as well as primary patient-derived SCC and BCC tumor samples ex vivo. We found that gas plasma treatment can induce apoptosis in vitro and in vivo and to modulate the inflammatory profiles of the skin tumor biopsies regarding the secretion of chemokines and cytokines as well as the expression of immune-related surface markers.

## 2. Results

### 2.1. Toxicity of Gas Plasma Treatment in Skin Cancer Cells In Vitro

**Figure 1:**
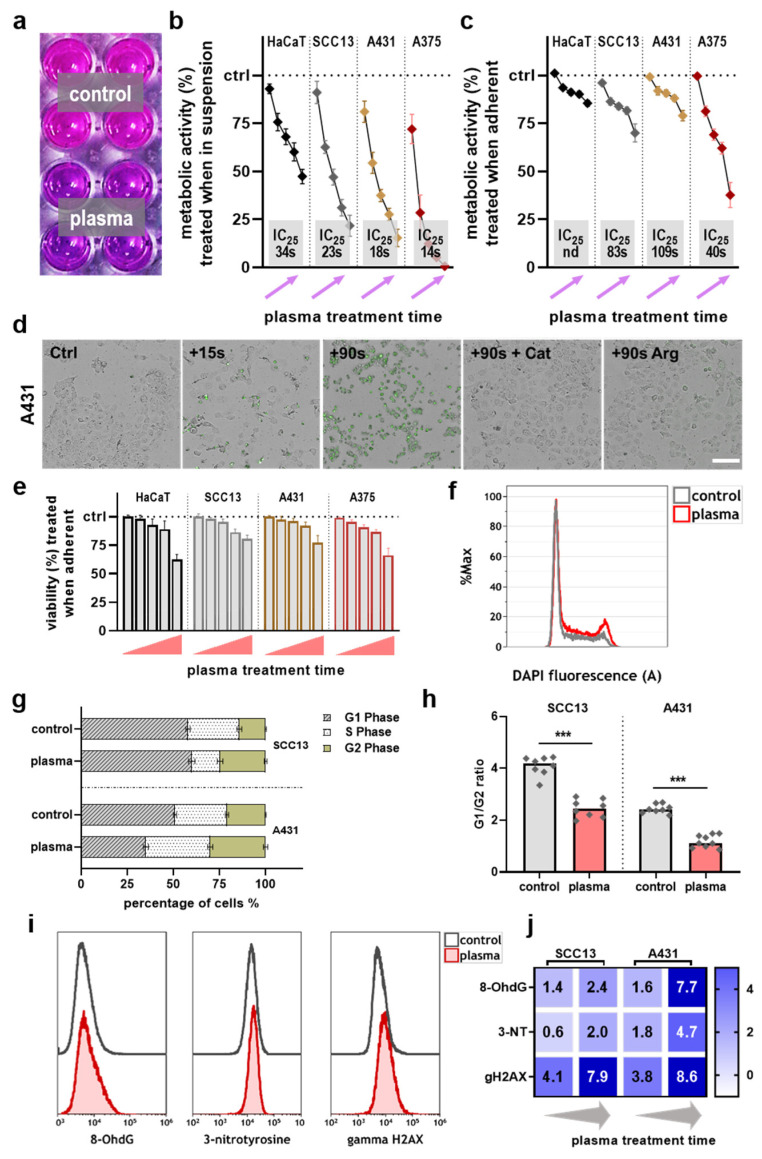
Gas plasma treatment in vitro: (**a**) image of the metabolic activity assay with higher (control) and lower (bottom) resazurin transformation, respectively; (**b**,**c**) quantitative analysis of gas plasma treatment effects in four cell lines exposed in suspension (**b**) or adherent (**c**) states, where grey boxes indicate IC_25_ values; (**d**) representative brightfield and fluorescence (caspase 3/7, green) overlay images of cells exposed to various conditions, catalase (cat) and argon gas treatment (Arg) served as controls; (**e**) viability of the four cell lines exposed in adherent state to several plasma treatment times (15 s, 30 s, 45 s, 60 s and 90 s); (**f**) representative overlay histogram of the DAPI intensity of control and gas plasma-treated SCC13 samples; (**g**) quantification of cell cycle phases in fixed and permeabilized DAPI-stained SCC13 and A431 cells after acquisition using flow cytometry and analysis of individual cell cycle phases using mathematical modeling according to the Michael H. Fox algorithm employed in the Kaluza analysis software; (**h**) G1/G2 ratios calculated from cell cycle data; (**i**,**j**) representative flow cytometry overlay histograms (**i**) and normalized quantification of three oxidative stress-related markers (**j**). Data were normalized to control and are displayed as the mean with SEM of three experiments. Statistical analysis was performed using *t*-test with *p* < 0.001 (***). Scale bar is 100 µm.

The toxicity of different doses of plasma irradiation was examined in four cell lines. A resazurin-based assay was used, which is indicative of total metabolic activity (Figure 1a). Two treatment modes were investigated. The first addressed toxicity in cell lines in suspension, while the second was performed in the cell lines that were allowed to adhere before treatment. An exposure time-dependent decline of metabolic activity was found in the former approach (Figure 1b). Based on these data and the calculated inhibitory concentration 25 (IC_25_), non-malignant HaCaT keratinocytes were less prone to gas plasma-induced metabolic activity reduction than the skin cancer cell lines. However, in the latter approach treating cell lines in adherent states, reduced abrogation in metabolic activity was identified (Figure 1c). Again, HaCaT cells’ resistance to gas plasma treatment was much greater than those of the other three skin cancer cell types. Due to the fact that the plasma treatment of cells in suspension is less relevant for the therapy of human disease, we focused on the stress responses of adherent plasma-treated cells in the following experiments. Thereby, the previous results were re-iterated by assessing the viability of cells based on caspase 3 and 7 activity and terminal cell death (Figure 1d). A comparable viability reduction was observed after gas plasma exposure across all cell types treated in adherent states (Figure 1e). By analyzing the different cell cycle phase in two tumor cell lines (Figure 1f), changes in the quantitative distribution of the different cell cycle phases were observed in both cell lines, which are indicative of cell cycle arrest (Figure 1g), as confirmed by the calculation of G1/G2 ratios (Figure 1h). The flow cytometry analysis of markers for oxidative stress (Figure 1i) subsequently showed overall treatment time-dependent elevated levels in gas plasma-treated skin cancer cells (Figure 1j), especially for phosphorylated (γ) histone 2A.X (γH2AX), suggesting oxidative stress-induced cell death.

### 2.2. Toxicity and Immunomodulation of Gas Plasma-Treated Skin Cancer Biopsies

**Figure 2:**
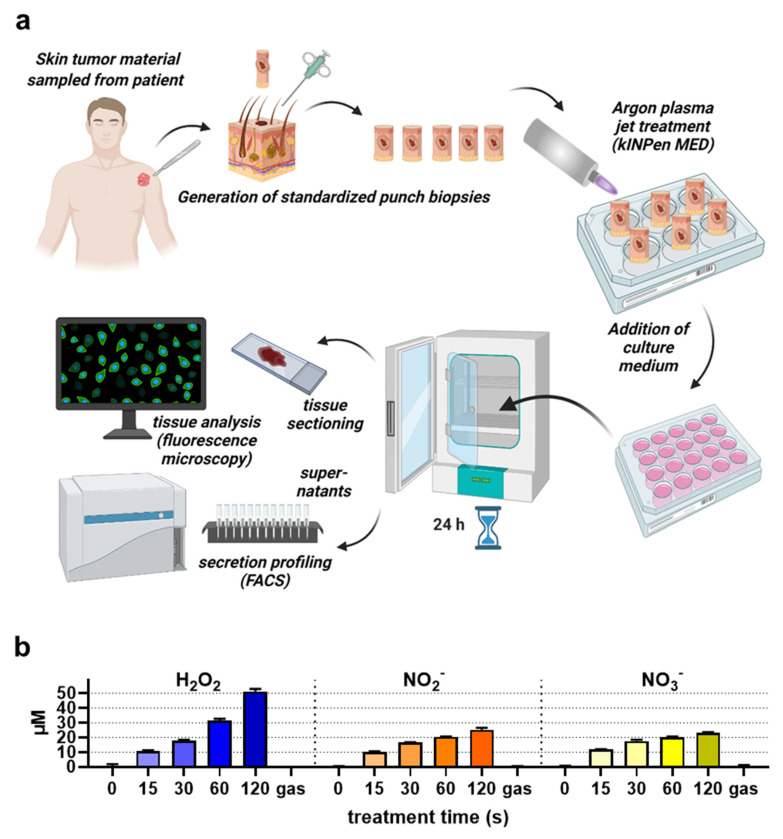
Human tumor tissue sampling and treatment scheme: (**a**) excised skin tumors were retrieved and used to generate punch biopsies of identical sizes. The punch biopsies were subsequently added to microtiter well plates for standardized gas plasma treatment. Afterward, cell culture medium was added to each well and the samples were incubated for 24 h, before supernatants were collected and tissues were cryo-sectioned and stained. Supernatants were analyzed using multiplex flow cytometry, and cryo-sections were stained with antibodies, followed by quantitative immunofluorescence imaging; (**b**) quantification of ROS (hydrogen peroxide, H_2_O_2_) and RNS (nitrite, NO_2_^−^; nitrate, NO_3_^−^) in plasma-treated liquids (200 µL of PBS). Gas refers to 120 s of exposure of the liquid with argon gas only (i.e., plasma = off).

**Figure 3:**
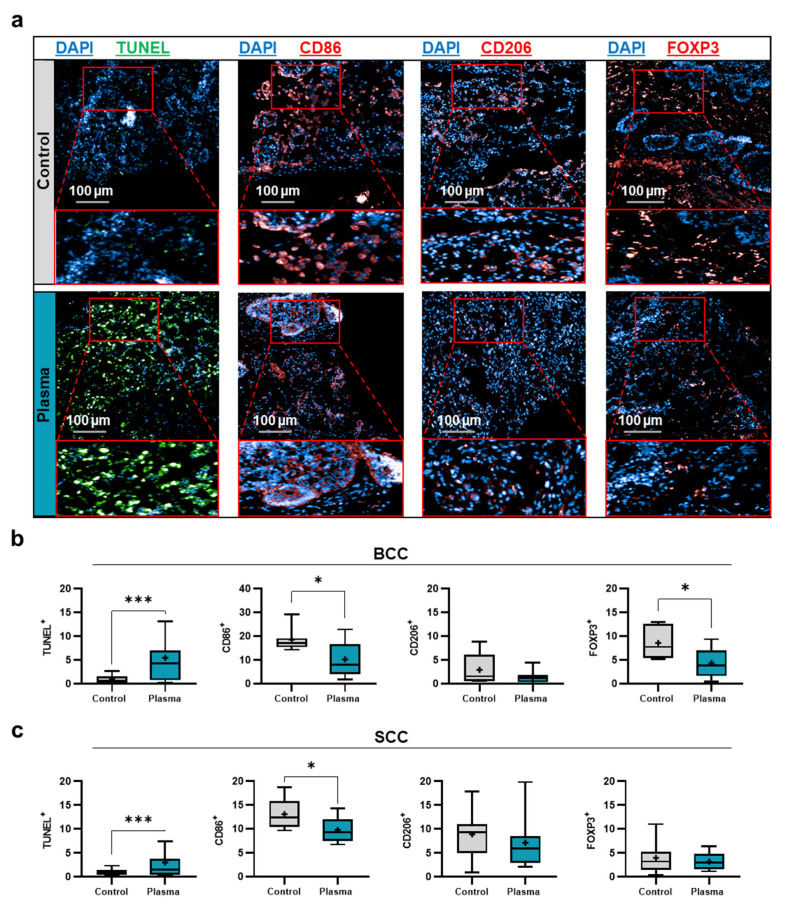
Immunofluorescence analysis: (**a**) representative immunofluorescence images of DAPI, TUNEL, CD86, CD206, and FOXP3 staining in gas plasma and argon gas (control)-treated BCC tissues; (**b**) quantitative imaging data for all markers in BCC samples; (**c**) quantitative imaging data for all markers in SCC samples. Data show boxplots from 6–8 patients, and statistical analysis was performed using *t*-test with *p* < 0.05 (*) and *p* < 0.001 (***). Scale bars are 100 µm.

**Figure 4:**
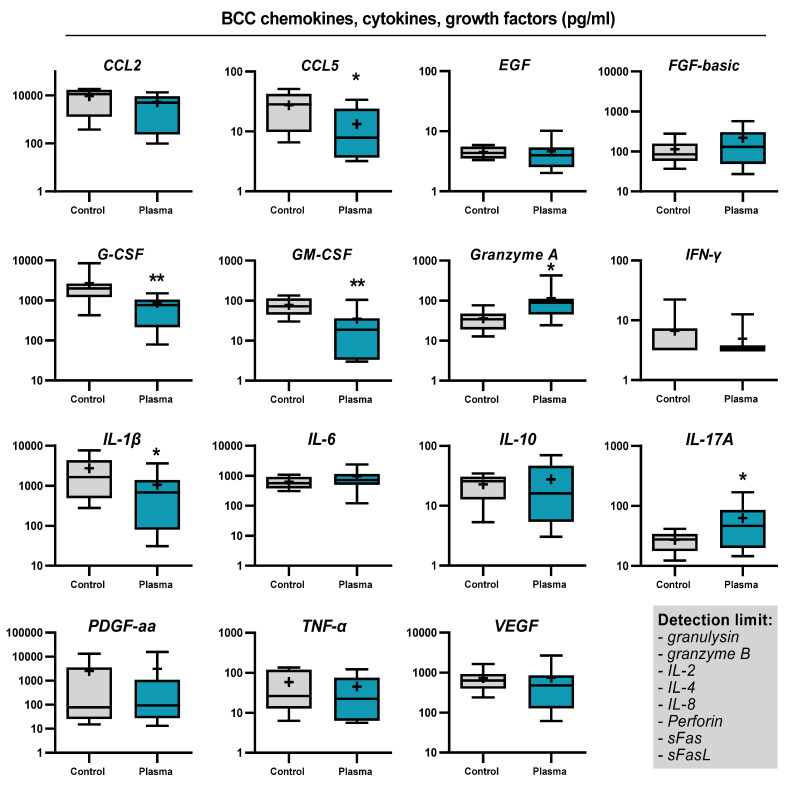
Secretion profile of BCCs: quantification of chemokines, cytokines, and growth factors in pg/mL in BCC tissue culture supernatants 24 h after exposure to gas plasma or argon gas. Data show boxplots from 6–8 patients, and statistical analysis was performed using *t*-test with *p* < 0.05 (*) and *p* < 0.01 (**).

**Figure 5:**
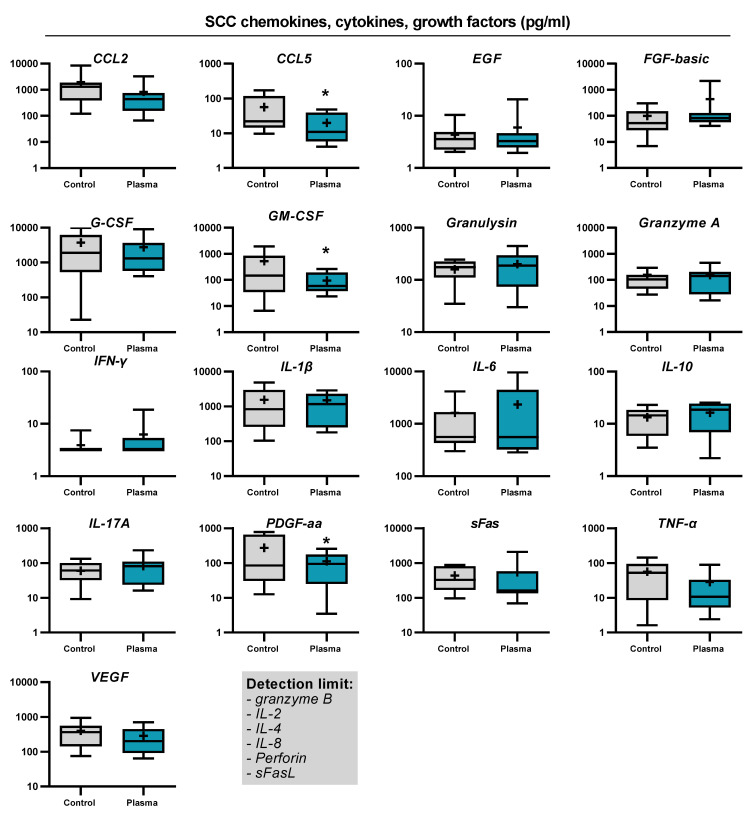
Secretion profile of SCCs: quantification of chemokines, cytokines, and growth factors in pg/mL in BCC tissue culture supernatants 24 h after exposure to gas plasma or argon gas. Data show boxplots from 6–8 patients, and statistical analysis was performed using *t*-test with *p* < 0.05 (*).

SCC and BCC skin cancer samples were collected from patients to generate biopsies of equal sizes and volumes, followed by gas plasma exposure ex vivo, incubation, and analysis of immunofluorescence tissue analysis and screening for soluble inflammatory markers in the tissue culture supernatants (Figure 2a). To verify that the kINPen used in this study generates reactive oxygen and nitrogen species as collectively described before [34], the stable and long-lived products hydrogen peroxide (H_2_O_2_), nitrite (NO_2_^−^), and nitrate (NO_3_^−^) were quantified in 200 µL of phosphate-buffered saline (PBS) after plasma exposure of different treatment times. Argon gas alone (applied at the maximum exposure time used for plasma treatment) served as the control in addition to untreated liquid (i.e., 0s). For all three types of oxidants investigated, their concentration increased in a plasma exposure time-dependent manner (Figure 2b). While the generation of H_2_O_2_ was nearly linear to the plasma treatment time, nitrite and nitrate were quickly generated during short plasma exposure but showed a slower rise for longer treatments. Tissues were sectioned and analyzed for apoptosis and the expression of several markers expressed by cells of the TME (Figure 3a). For BCC, gas plasma exposure significantly increased the percentage of apoptotic cells, while the expression of CD206, a marker of tumor-supportive M2 macrophages, remained unchanged (Figure 3b). Interestingly, a significantly lower expression was found for CD86, a molecule associated with professional antigen-presenting cells, and FOXP3, a transcription factor of regulatory T cells (T_reg_). Elevated levels of apoptosis were also found in ex vivo gas plasma-treated patient-derived SCC tissue, while CD206 remained unchanged as in BCC (Figure 3c). Similar to BCC, CD86 was significantly reduced, however, by contrast, the T_reg_ numbers remained unchanged. The immunomodulatory effects of gas plasma treatment on primary skin cancer samples were further investigated by quantifying more chemokines, cytokines, and growth factors released into the culture medium during the incubation period. For BCCs, a significant reduction in CCL5, G-CSF, GM-CSF, and IL-1β was observed, while granzyme A and IL-17A release was significantly elevated (Figure 4). In SCC samples, three targets were significantly regulated, namely CCL5 and GM-CSF which were upregulated as observed in the BCC samples, and PDGF-aa being reduced (Figure 5). These results suggest gas plasma treatment to confer toxicity in SCC and BCC and to have an immunomodulatory role.

## 3. Discussion

The capacity of cold physical plasma employed in medical gas plasma technology in killing tumor cells was predicted via the direct induction of ROS in tumor cells and the inhibition of tumor cells’ defense mechanism and antioxidant mechanisms [35,36]. This study demonstrated gas plasma-induced toxicity in vitro and in patient-derived skin cancer samples ex vivo together with immunomodulatory effects.

In vitro, a gas plasma exposure time-dependent and selective toxicity was observed. In general, the selectivity of gas plasma treatment is a matter of debate and depends on the type of cell line used as comparator [9]. Hence, both selectivity [37,38] and non-selectivity [39,40] have been observed. Our findings of cell cycle arrest underline previous findings as well [41,42]. H2AX phosphorylation following gas plasma treatment has also been described before [43,44], albeit it should be noted that γH2AX induction is a consequence of apoptosis rather than directly short-lived ROS-mediated DNA double-strand breaks [45]. Pro-apoptotic effects of gas plasma in three-dimensional tumor tissues have been observed several times using, for instance, in vivo tumors [46,47] and tumor animal models [48,49]. However, regarding patient-derived skin tumor biopsies, we here describe gas plasma-induced apoptosis in cutaneous SCC and BCC. For oral SCC, gas plasma-induced apoptosis using the kINPen plasma jet has been reported before [50].

Gas plasma treatment of skin cancer tissues changed the expression and release of immuno-related molecules, respectively. CD86 is readily expressed by professional antigen-expressing cells to support T cell activation and was found to be decreased in gas plasma-treated SCC and BCC tissue samples. However, CD86 also is readily expressed on tumor cells [51,52,53], for reasons which to date remain unknown. High CD86 has been linked to unfavorable prognosis in different types of tumors [54,55]. CD206 remained unchanged in our study. The molecule is a M2a macrophage polarization marker (also called tumor-associated macrophage (TAM)) [56] and is associated with disease severity in BCC [57]. FOXP3-expressing T_regs_ are also a poor prognostic marker in BCC as well as SCC [58,59]. Although realistic immunodynamics, including extravasation and the infiltration of new leukocytes, is not reflected in our ex vivo model, our data at least suggest that gas plasma treatment also has an immunological dimension. The findings in tissue-cultured supernatants underlined this. The changes in BCC samples were more pronounced compared to SCC after gas plasma treatment. Both had a marked downregulation of CCL5 (RANTES) in common. There are many sources of CCL5 in skin cancer, for instance, dendritic cells, macrophages, cancer-associated fibroblasts, keratinocytes, mast cells, and tumor cells [60,61,62]. CCL5 serves as chemoattractant for lymphocytes and promotes tumor-supportive T_H_2 polarization [63]. The molecule is associated with poor prognosis in many types of cancers [64,65,66]. In BCC, but not SCC samples, gas plasma stimulated elevated levels of IL-17A. This was probably due to the higher baseline expression of IL-17A in BCC that is associated with a favorable prognosis [67]. GM-CSF levels significantly declined in SCC and BCC following gas plasma exposure. GM-CSF has pleiotropic roles in tumor biology, and expression is elevated in SCC [68], while at the same time, it has also been used as a therapeutic agent in non-melanoma skin cancer [69].

It is widely assumed that ROS are the main drivers of gas plasma-mediated tumor cell toxicity, especially in vitro [24]. Regarding the significance of ROS for tissue-mediated effects, less evidence is available. This is because tools that are capable of detecting ROS in tissues are generally scarce. Recently, we showed for the first time the direct oxidation of plasma-derived ROS into human subcutaneous tumors grown in immunodeficient mice using a luminescent probe [70]. In a human head and neck cancer patient, changes in the microcirculation were directly observed after plasma treatment [71], pointing to a role of fast effectors such as ROS and/or electric fields. Regarding the latter, mounting evidence suggests that electric fields modulated in gas plasma sources are in contact with targets [72,73]. Final evidence and models are awaited to distinguish the role of different effectors in the gas plasma treatment of tissues. This includes the types of species dominating biological effects in tissues. While in plasma-treated liquids [74] and in vitro, depending on the feed gas admixture used, the dominant presence of a few long-lived species were described [75], the main types of ROS reaching the target tissue at sufficient depth and quantity as well as the putative secondary redox chemistry remains to be elucidated. The physics and redox chemistry of the kINPen in the plasma gas phase is, by contrast, very well explored [76]. 

## 4. Materials and Methods

### 4.1. Cell Culture

The human skin epidermoid squamous cell carcinoma cell line A431, the human epidermal squamous carcinoma cell line SCC-13, the human melanoma cell line A375, and the human non-malignant HaCaT keratinocyte cell line were grown in fully supplemented (10% fetal bovine serum, 1% penicillin/streptomycin, and 2% glutamine) Roswell Park Memorial Institute (RPMI) 1640 medium (Pan-Biotech, Aidenbach, Germany). All cell lines were passaged twice per week and maintained under standard culture conditions (37 °C, 5% CO_2_, 95% humidity). For experiments, 1 × 10^5^ cells were seeded per well of a 24-well plate (NUNC, Roskilde, Denmark) in 500 µL of fully supplemented cell culture media.

### 4.2. Primary Skin Tumor Material

Study approval was received from the University of Duisburg-Essen (ethical committee of the University of Duisburg-Essen) under the Institutional Review Board protocol number 12-4961-BO. Patient samples were retrieved upon informed consent to perform the experiments and anonymously publish the data. Primary tumor material from human cutaneous squamous cell carcinoma (SCC) and basal cell carcinoma (BCC) was collected from ten patients each during surgery. Subsequently, 3 mm punch biopsies (without hampering histological diagnosis) were generated for each tissue in the laboratory to generate skin cancer tissue samples for further experiments with a standardized size and volume. The biopsies were added to customized conical receptacles, which were added to 96-well holders to ensure a standardized location and maintain their upright position.

### 4.3. Gas Plasma Exposure

The atmospheric pressure plasma jet kINPen (neoplas GmbH) was utilized for the in vitro experiments. The ambient air conditions were not monitored as it was recently shown that its role in the kINPen plasma jet’s tumor toxicity is minor [14,77]. For in vitro experiments, the argon feed gas flowed through a capillary (inner diameter 1.6 mm) with 1.5 standard liters per minute and plasma was ignited by a radio frequency voltage (1.1 MHz/2–6 kV, peak to peak, non-pulsed). A motorized and computer-controlled xyz-table (CNC, Germany) was used to hover the gas plasma jet over the central position of each well for different dwell times (15 s, 30 s, 45 s, 60 s, and 90 s). The exposure distance from the nozzle to the liquid target (as common in cell culture experiments) was 15 mm. The main reactive species being generated in the kINPen plasma gas phase and treated liquids have been thoroughly described before [76,78,79]. As a control, the samples were exposed to argon gas alone. Liquid evaporation was compensated for by adding predetermined amounts of double-distilled water immediately following the treatment. For the treatment of primary human skin biopsies, the atmospheric pressure argon plasma jet kINPen MED (neoplas MED, Greifswald, Germany) was used. The jet is accredited as medical product class IIa in the European Union for dermatological applications, especially wound healing [80], but it has not yet received licensing for cancer treatment. The gas plasma was generated at a frequency of 1 MHz and using five standard liters per minute of argon gas with an effective power of 1 W. The samples received gas plasma or argon gas treatment for 120 s at a distance of 1 cm between the capillary and the tumor tissue, so that the jet was touching the tissue surface (conductive mode). The jet was operated at body temperature and does not cause thermal damage to treated tissues [80]. Following plasma treatment ex vivo, the tumor samples received fully supplemented cell culture medium. After 24 h of incubation under standard culture conditions, the supernatants were collected and stored at −80 °C until analysis. Then, the tissue samples were embedded in OCT, added to disposable molds, snap-frozen using liquid N2, and stored at −80 °C.

### 4.4. Metabolic Activity

Twenty hours after exposure, resazurin (7-Hydroxy-3H-phenoxazin-3-one 10-oxide) was added to the cells (final concentration: 100 µM). Metabolically active cells transformed the non-fluorescent resazurin into fluorescent resorufin. After 4 h of incubation in the cell culture incubator, the well plate was transferred to a multimode plate reader (F200; Tecan, Mennedorf, Switzerland), and fluorescence was determined at λ_ex_ 535 nm and λ_em_ 590 nm. The data were background-subtracted using wells containing fully supplemented cell culture medium without cells and resazurin and normalized against the argon gas-treated control, in which the plasma of the jet has been switched off. Pilot experiments confirmed no effect of the argon gas treatment compared to untreated cells.

### 4.5. Apoptosis Detection

Twenty-four hours after exposure, CellEvent caspase 3/7 detection reagent (ThermoFisher, Bremen, Germany) was added, and the cells were cultured for another 30 min. The dye quickly entered cells and contained a fluorescent moiety with high affinity and fluorescence once bound to DNA but only after it has been released by enzymatic activity performed by the caspases 3 and 7. Afterwards, the plate was imaged using fluorescence microscopy (Operetta CLS; PerkinElmer, Hamburg, Germany) by the acquisition of brightfield and fluorescence (λ_ex_ 475 nm and λ_em_ 525 nm) images, and quantitative image analysis (Harmony 4.9 software; PerkinElmer, Germany) was performed.

### 4.6. Flow Cytometry

Twenty-four hours after exposure, cells were detached using accutase and subsequently fixed and permeabilized. For cell cycle analysis, DAPI (4′,6-Diamidino-2-phenylindol) was added (final concentration: 10 µM) and incubated at 4 °C for 1 h. After washing, cells were resuspended in FACS buffer and acquired using flow cytometry (CytoFLEX LX; Beckman-Coulter, Krefeld, Germany). Alternatively, fixed cells were stained using fluorescently labeled monoclonal antibodies targeting 8-OHdG (8-Hydroxy-2′-deoxyguanosine), 3-NT (3-nitrotyrosine), and γH2AX (phosphorylated histone 2A family member X). After 1 h of incubation at 4 °C, cells were washed and acquired using flow cytometry. Data analysis was performed utilizing Kaluza 2.1 software (Beckman-Coulter, Germany). The quantification of cells in the G1, S, and G2 phases of the cell cycle was done using mathematical modeling based on the Michael H. Fox algorithm [81].

### 4.7. Supernatant Analysis

To detect inflammatory mediators, multiplex analysis was performed using bead-based assay kits (BioLegend, Amsterdam, The Netherlands) to simultaneously assess several cytokines, chemokines, and growth factors according to the manufacturing company’s instructions. To achieve this, the supernatant collected from the tumor tissue cultures was incubated with antibody-coated beads. After washing, the addition of streptavidin beads, and another washing step, the analytes’ mean fluorescent intensity was determined using a flow cytometry device (CytoFLEX S; Beckman-Coulter, Germany) and the LegendPlex Software (BioLegend, The Netherlands). For absolute quantification, 5-log standard curves were generated for each of the targets.

### 4.8. Tissue Immunofluorescence Analysis

Tissue material was sectioned using a cryotome to retrieve 7 μm-thick sections placed on the microscopy slides (SuperFrost Plus Adhesion slides; ThermoFisher, Germany). The tissues were fixed using 4% paraformaldehyde for 10 min at room temperature. To retrieve the antigens of interest and increase permeability, the tissues were placed into sodium citrate and Triton X-100 solution for 20 min. Washing with PBS was performed three times, each time lasting 5 min, between different staining stages. Then, the tissues were blocked in 3% BSA solution in PBST for 30 min to prevent the non-specific binding of antibodies which were added afterwards. For staining, anti-CD86 (Abcam: Ab53004), anti-CD206 (Novus: NBP1-90020), and anti-FOXP3 (Novus: NB100-39002) antibodies were used and incubated in PBST solution containing 1% BSA overnight at 4 °C. After washing, appropriate Alexa Flour 647-conjugated secondary antibodies (Thermo Fisher, Germany) were added. Then, TUNEL staining was performed according to the manufacturer’s instructions (Sigma-Aldrich, Taufkirchen, Germany) for frozen tissues to identify apoptotic cells. Finally, nuclei staining was performed by using DAPI. The slides were imaged using the fluorescence microscope’s 20x air objective (NA = 0.4) (Operetta CLS). The bandpass centers for fluorescence capturing were λ_ex_ 365 nm and λ_em_ 450 nm for detecting DAPI, λ_ex_ 475 nm and λ_em_ 525 nm for detecting TUNEL-stained apoptotic cells and λ_ex_ 630 nm and λ_em_ 700 nm for detecting the antibody-labeled cells. Image acquisition, analysis, and quantification were performed by using Harmony 4.9 software.

### 4.9. Reactive Species Analysis

To quantify the amounts of the long-lived oxidants H_2_O_2_, NO_2_^−^, and NO_3_^−^, known products of short-lived ROS/RNS generated in the plasma gas phase [34], 200 µL of PBS were exposed to the kINPen as described for the treatment of cell cultures. After evaporated liquid was compensated for, analysis was performed as previously described in detail [78]. 

### 4.10. Statistical Analysis

Data graphing and statistical analysis were conducted using Prism 9.2 (GraphPad Software). The *T*-test was used to compare the difference between two groups statistically. The determination of IC_25_ values was done using nonlinear regression analysis against log-2 transformed exposure times. The confidence interval was 95% for all tests, and statistically significant differences were marked with * (*p* < 0.05), ** (*p* < 0.01), or *** (*p* < 0.001).

## 5. Conclusions

Our proof-of-concept study shows cytotoxic and immunomodulatory effects of gas plasma jet treatment in patient-derived BCC and SCC samples, calling for future clinical exploration of this technology in non-melanoma skin cancer treatment. At the same time, substantial efforts are needed to decipher the gas plasma-derived reactive species chemistry necessary to induce the tumor toxic effects and to elucidate potential optimization routes of this technology to serve as an adjuvant oncological therapeutic option in the long term.

## Data Availability

The underlying data are available from the corresponding author upon reasonable request.
